# Commentary: Synergistic AI-resident approach achieves superior diagnostic accuracy in tertiary ophthalmic care for glaucoma and retinal disease

**DOI:** 10.3389/fopht.2025.1749411

**Published:** 2025-12-19

**Authors:** Kevin Williams-Gaona, Rosa Corro

**Affiliations:** 1 Caja de Seguro Social (CSS) Dr. Hugo Spadafora, Colón, Panama; 2 Department of Research, Mayo Clinic Florida, Jacksonville, FL, United States

**Keywords:** artificial intelligence, clinical training, diagnostic workflow, medical education, ophthalmology

Camacho-García-Formentí et al. present an impressive demonstration of how a synergistic collaboration between artificial intelligence systems and resident physicians can improve diagnostic accuracy in glaucoma and retinal disease. Not only do they show that an AI-resident partnership can outperform either one alone, they also demonstrate something that many groups struggle to capture: how these systems behave in a real tertiary-care environment. Anyone who has worked in a busy ophthalmology service knows that elegant results on paper are one thing, and making them coexist with high patient load, mixed pathology, and time pressure is another. Their team managed both.

Even though the combined approach was the top performer, the study also shows that AI on its own outperformed first-year residents across several key measures, including higher accuracy in glaucoma suspect classification (88.6% vs. 82.9%) and much higher sensitivity for retinal disease (76% vs. 52%, and 100% for high-risk findings). AI’s CDR estimates also tracked more closely with expert measurements than those of residents (r = 0.728 vs. 0.538), although the system could only evaluate CDR in 61.6% of patients because of image-quality issues. That detail matters: even when an algorithm performs well, real-world imaging is variable, and someone still needs to handle the cases it cannot process.

As we read through their results, a central question emerged. We talk a lot about “human in the loop” AI, and it’s usually framed as a reassuring idea. The algorithm assists, but the clinician remains in control. That logic works today, when clinicians have years of pattern recognition behind them. But what happens when the human entering the loop has had fewer chances to build the very skills the loop depends on?

Ophthalmology is built on repetition. Residents grow by seeing normal variants, borderline OCTs, unusual discs, cases that fooled everyone for a moment, and even the occasional false alarm. These encounters are not random; they form the “texture” of training. And with an incorporated system of AI reaching 76% sensitivity overall and up to 100% for high-risk findings, the educational risk may shift toward reduced cognitive effort. The stakes of being wrong feel lower, and it becomes easier for trainees to lean on the model’s output. That subtle shift is enough to reshape how clinical judgment develops.

That picture shifts even more if these systems begin to be used simultaneously or as a first-pass screening tool, in favor of the higher accuracy demonstrated by a synergistic approach. Current discussions about glaucoma care already consider AI-based screening and triage as likely components of routine workflows (1, 2). For example, if AI becomes very reliable at identifying early glaucoma or prioritizing retinal findings and begins filtering or labeling before a trainee even looks at an image, residents may start encountering a narrower slice of disease, mostly the ambiguous or the highly complex. That sounds ideal at first, but exposure to the full spectrum is what builds confidence in calling something “normal” or “stable,” which is just as important as diagnosing pathology. And when AI makes the right call 90% of the time, trainees might, without meaning to, start deferring to the algorithm instead of forming their own mental map first.

Of course, this future is not inevitable. There is room for deliberate design. AI systems could be built with “teaching modes” that intentionally route uncertain or instructive cases to trainees before any automatic labeling. They could generate sets of high-yield comparisons, or highlight regions of low model confidence so residents learn where humans still outperform machines. Early work in explainable AI and recent discussions on supervised integration suggest that these kinds of interactions could become a genuine educational asset (3).

But none of that will happen if training is not part of the AI conversation from the beginning. The article by Camacho-García-Formentí et al. shows how well AI can support clinical care right now. What we hope to add is that implementation planning should also consider how residents will grow inside these new systems. The “human in the loop” model only works if the human entering the loop is well prepared, and that preparation depends on protecting opportunities for independent clinical reasoning.

Their study opens the door to improving accuracy and workflow efficiency. The next step is making sure the same progress strengthens, rather than narrows, the education of future ophthalmologists.
